# Resident wild koalas show resilience to large-scale translocation of bushfire-rescued koalas

**DOI:** 10.1093/conphys/coac088

**Published:** 2023-01-28

**Authors:** Julian E Beaman, Connor Mulligan, Claire Moore, Dana Mitchell, Edward Narayan, Karen Burke da Silva

**Affiliations:** College of Science and Engineering, Flinders University, Bedford Park, South Australia 5042; College of Science and Engineering, Flinders University, Bedford Park, South Australia 5042; College of Science and Engineering, Flinders University, Bedford Park, South Australia 5042; Kangaroo Island Wildlife Park, 4068 Playford Hwy, Duncan, South Australia 5223; Kangaroo Island Koala & Wildlife Rescue Centre, 4068 Playford Hwy, Duncan, South Australia 5223; School of Agriculture and Food Sciences, The University of Queensland, Lawes, Queensland 4343; College of Science and Engineering, Flinders University, Bedford Park, South Australia 5042

**Keywords:** wildlife management, wildlife health, Wildlife conservation

## Abstract

Wildlife translocation is increasingly utilized as a conservation management action, to mitigate the immediate negative effects of habitat loss and fragmentation (e.g. from land clearing or bushfires). Previous research has shown that stress responses can help or hinder survival in translocated wildlife and determine the efficacy of translocation as a conservation action. Yet these translocated animals are only one side of the equation, with translocation also potentially impacting the animals in the recipient population. We measured physiological markers of stress (faecal cortisol metabolite concentrations and neutrophil-lymphocyte ratios) and assessed health condition in a wild koala population one year after a major translocation of bushfire-rescued koalas on Kangaroo Island. We expected to find a high population density at the site (>0.75 koalas per hectare) and that resident koalas would show signs of chronic stress and ill health as a result of territorial conflict over food trees and reproductive opportunities. In contrast, we found that only one-fifth of the population remaining at the site were translocated koalas. The overall population density was also much lower (0.21 koalas per hectare) than anticipated. With no evidence of mass mortality at the site, we suggest that the majority of translocated koalas dispersed away from the site. Our stress marker measurements did not differ between the wild koalas and a sample of captive (non-display) koalas at the nearby Kangaroo Island Wildlife Park and were generally low compared to other studies. Veterinary examinations found that most koalas were in good body condition with very few diagnostic indicators of systemic ill health. Overall, our results suggest that, if there is adequate landscape-scale habitat connectivity and opportunity for dispersal, translocated koalas are likely to disperse from the site of release, with limited impacts on recipient koala populations at translocation release sites.

## Teaser

Koalas are often transported between habitats in an attempt to mitigate the negative impacts of bushfires on local populations. We found that the koalas already living at a release site showed very few signs of lasting stress or ill-health following a large-scale translocation of bushfire-rescued koalas on Kangaroo Island.

## Introduction

The translocation of wildlife has become an important management action in the conservation of threatened species (Weeks *et al.*, 2011; Berger-Tal *et al.*, 2020). The process of translocation, however, induces stress in the animals being moved (Teixeira *et al.*, 2007) and potentially also those already occupying the areas of habitat (the recipient population) into which translocated animals are released. In the short term, acute stress can facilitate adaptive behavioural and physiological responses (allostasis; McEwen, 1998), which improve survival probability after translocation (Cabezas and Moreno, 2007). Yet over the longer term, chronic stress (i.e. distress) can negatively affect survival and reproductive success, thereby reducing the efficacy of translocation as a conservation action (Dickens *et al.*, 2010). Measurements of physiological markers of stress (Narayan *et al.*, 2013; Davis and Maney, 2018) can help guide translocation practice and evaluation of translocation success (Cabezas and Moreno, 2007; Franceschini *et al.*, 2008). To date, efforts to integrate physiological measurements into translocation studies have focused on the animals being translocated (Tarszisz *et al.*, 2014). What remains poorly understood, however, is how the translocation of new individuals into an existing population affects the individuals in the recipient population (Flesch *et al.*, 2020; Langridge *et al.*, 2021). The current lack of data on the potential health impacts of translocation on recipient populations is a barrier to objective assessments of the utility of translocation as a conservation intervention.

Koalas (*Phascolarctos cinereus*) are one such species for which translocation has long featured as a conservation management action (Menkhorst, 2008). In the east of Australia, where koalas are listed as endangered under federal law (Department of Agriculture Water and the Environment, 2022), translocation is used to mitigate the impacts of land clearing and urbanization (Beyer *et al.*, 2018). Conversely, in southern Australia, translocation has previously been used to manage the impacts of koala over-abundance on native vegetation communities (Menkhorst and Rylah, 2017; Whisson and Ashman, 2020). Major bushfires are also increasing the number of rescued koalas admitted to wildlife hospitals (Dunstan *et al.*, 2021; Parrott *et al.*, 2021), with rehabilitated koalas commonly needing to be translocated to unburnt (or moderately burnt) habitat that already supports koalas when their return to severely burnt habitat is not an option. There is clearly a pressing need for scientific assessments of the impacts of translocations on the health and physiology of koalas, both the individuals being translocated, as well as the population into which translocation has occurred, to determine the success of translocation as a conservation action.

One of the main challenges facing koalas at translocation release sites is to maintain a territory with sufficient food resources and mating opportunities. Koalas almost exclusively eat eucalyptus leaves and selectively forage from a subset of potential food trees based on the nutrient quality and toxin content of leaves (Hindell and Lee, 1987; Moore and Foley, 2000; Moore and Foley, 2005; Melzer *et al.*, 2014). Hence, competition for territories will vary depending on the availability of quality food resources in the environment. Resource availability does appear to impact territory size, with koalas occupying home-ranges sometimes more than 100 ha in sparsely treed habitats in north-eastern Australia and less than 1 ha in densely forested areas in southern Australia (Davies *et al.*, 2013a, Ellis *et al.*, 2002, Whisson *et al.*, 2016). Male koalas are more likely than females to engage in aggressive interactions in competition for mates (Melzer *et al.*, 2010), while females are known to tolerate closer interactions with other female and male koalas (Ellis *et al.*, 2015). If competition for food resources and aggressive encounters increase after translocation, there is potential for translocation to lead to chronic stress and poor health outcomes in koalas that remain at the translocation site.

Chronic stress can lead to a range of health complications, including increased susceptibility to infectious diseases, organ disfunction and reproductive failure (Sapolsky *et al.*, 2000; Dhabhar, 2009; Martin, 2009; Whirledge and Cidlowski, 2010). In koalas, chronic stress potentially increases the severity of endemic infectious diseases, such as chlamydia and koala retrovirus (KoRV), but direct evidence is currently lacking (Narayan and Williams, 2016; McCallum *et al.*, 2018). Calcium oxalate nephrosis is also a co-morbidity of distressed koalas but the cause-and-effect relationship between stress and renal disfunction is unclear (Gonzalez-Astudillo *et al.*, 2019, Narayan and Williams, 2016, Speight *et al.*, 2018).

There are two commonly measured markers of stress applied to wild mammal populations. One approach is to measure the concentration of corticosteroid metabolites in faecal samples (Narayan *et al.*, 2013), and the other is relative counts of white blood cells (WBCs) (Davis and Maney, 2018). The endocrine mechanisms underlying the mammalian stress response have been extensively reviewed elsewhere (Reeder and Kramer, 2005). Briefly, exposure to a stressor stimulates the hypothalamo–pituitary–adrenal (HPA) axis, releasing adrenocorticotropic hormone (ACTH) from the anterior pituitary gland, which in turn stimulates the secretion of glucocorticoids (cortisol and/or corticosterone) from the adrenal cortices. Circulating glucocorticoids are then metabolized into constituent metabolites and excreted in the faeces over time, meaning that measurements of FCM provide a measure of the background level of stress hormones circulating in the blood in the days prior to sampling. Numerous challenges to the accurate detection and interpretation of hormone metabolite concentrations in faeces have been highlighted, including the potential for cross-reactivity of enzyme immunoassays (EIA) and the effects of diet, sex and genetic variation in metabolism (Goymann, 2012; Pribbenow *et al.*, 2017). While these issues remain a challenge, experimental validation of EIA assays for cortisol have been developed in koalas in which the downstream effects of a pharmacological stress test (administration of ACTH) were detected in measured FCM concentrations after 1–2 days and for up to at least 9 days (Narayan *et al.*, 2013).

The other commonly measured stress marker in mammals is the relative counts of WBC (leukocytes), specifically the ratio of neutrophils to lymphocytes (reviewed in Davis and Maney, 2018, Davis *et al.*, 2008). Briefly, the stress response via the HPA axis and the secretion of glucocorticoids has the effect of simultaneously increasing the number of neutrophils (neutrophilia) and decreasing the number of lymphocytes (lymphopenia or lymphocytopenia). Because stress alters the abundance of neutrophils and lymphocytes in opposite directions, high neutrophil to lymphocyte (N:L) ratio acts as an additional indicator of stress. A high N:L ratio can also be an indicator of neutrophilia secondary to an inflammatory process or systemic infection. Hence, the absolute values of measurements of stress markers such as N:L ratios and FCM concentrations can be challenging to interpret due to potential underlying disease burden, as well as biological variation in stress markers associated with age, sex, circadian rhythms and seasonality (i.e. breeding season) (Santamaria *et al.*, 2021). The implication is that measuring markers of physiological stress in combination with physical assessments of body condition, disease prevalence, systemic health and reproductive status serves as a practical and holistic way to assess the potential impacts of translocation on wild koala populations.

Here, we report on a study that aimed to assess the outcomes of a large-scale release of rehabilitated koalas on Kangaroo Island, South Australia, in the wake of the Black Summer bushfires of 2019/2020. Tens of thousands of koalas perished during the bushfires on Kangaroo Island, which reduced the population by 80–90% from an estimated 50 000 before the fire (Molsher, 2017) down to 5000–10 000 after the fire (*unpublished data*, South Australian Department for Environment and Water). Understanding the health and resilience of the remaining koalas on Kangaroo Island is crucial for future management of this chlamydia-free population (Fabijan *et al.*, 2019), which is a status that may see this population become a bastion for the species. During the bushfires, more than 600 rescued koalas were brought into the Kangaroo Island Koala Rescue Centre. Many were severely injured and required humane euthanasia whereas those able to be rehabilitated were cared for until they were ready for release back into the wild (Dunstan *et al.*, 2021). The intensity and extent of the fires in many areas of Kangaroo Island were such that it was not feasible to release these koalas back into the burnt areas in which they had previously lived, and many rescued koalas were also of unknown origin. As a result, the best option was to release rehabilitated koalas into areas of unburnt koala habitat on private land.

We studied the wild koala population at a translocation site more than 1 year after the major release of rescued koalas (90 individuals) into the area (108 ha, see *Study site* below for detailed description). First, we estimated the population density of koalas (resident and rescue-release) remaining in the habitat area. We anticipated that some of the koalas released into the area would have dispersed via habitat corridors into the surrounding landscape, but that many would remain at the site causing population density to increase to high levels (more than 0.75 koalas per hectare). We then assessed whether koalas remaining at the release site (resident and released) were showing evidence of chronic stress, poor health or physical injury. To do so, we measured FCM concentrations and N:L ratios, in combination with physical examinations of body condition and haematological assessments of systemic health. In addition to our measurements in wild koalas at the translocation site, we also measured the same variables in a cohort of koalas that had remained captive at the wildlife park since the rescue. Our measurements in a captive population serve as useful comparison with the wild population since the koalas belong to the same genetic population for which there have been no previous measurements of FCM concentrations, and only one study that measured N:L ratios (Fabijan *et al.*, 2020b). Because the captive koalas had been kept away from public viewing and had permanent access to food, water and shelter, we expected that indications of chronic stress in the wild population would be evidenced by higher levels of FCM concentrations, higher N:L ratios and poorer indicators of body condition and systemic health compared to the captive population.

## Methods

### Ethics approvals

All aspects of the present study were approved by the Flinders University Animal Welfare Committee (approval number 4222–10).

### Study site

Wild koalas were sampled at a study site in the central area of Kangaroo Island, South Australia (−35.77722, 137.22305), comprising 108 hectares of continuous, mixed forest of preferred koala food tree species (*Eucalyptus obliqua* and *E. baxteri*). The site did not burn during the Black Summer bushfires of 2019/2020, but the fire front came to within 2 km of the site. The study site had an unknown density of resident koalas at the time of translocation release, but the vegetation showed no evidence of over-browsing by koalas. Koala over-browsing is typically observable at a population density more than 0.75 koalas per hectare (Delean and Prowse, 2019). A comprehensive literature review of koala translocations shows that survival is greatest when koalas are released into habitat areas greater than 100 ha (Menkhorst and Rylah, 2017). Importantly, while the release site is only marginally larger than 100 ha, it is well connected to other unburnt koala habitat in the broader landscape, with riparian vegetation and linear strips of remnant vegetation retained along farm paddock fence-lines providing dispersal corridors for koalas.

### Population density survey

Koala population density at the study site was estimated using a two-count mark-resight technique (Caughley and Sinclair, 1994; Masters *et al.*, 2004). The study area was divided into 5-hectare areas within which two experienced observers independently searched for all koalas in the area for a maximum of 2 h. When a koala was observed, a mark was placed at the base of the tree. If the second observer also spotted the same koala, they would record their observation as a ‘resighting’ if there was a mark at the base of the tree. The observations from both observers were used to estimate population size with the following formula:


$$ Y=\frac{\left(B+{S}_1+1\right)\left(B+{S}_2+1\right)}{B+1}\hbox{--} 1, $$

where *B* is the number of koalas seen by both observers, *S_1_* is the number of koalas seen by observer 1 but not observer 2 and *S_2_* is the number seen by observer 2 but not observer 1.

### Sample size and koala capture

A total of 30 koalas were sampled, 13 males and 17 females (all adults except for two independent sub-adults, based on tooth-wear age classification). Of these, 10 (4 males and 6 females) were resident in captivity (but not on display, except one male) at the Kangaroo Island Wildlife Park nearby (<1 km) the bushland study site. The other 20 koalas (9 males, 11 females) were sampled from the wild population at the study site between June and September 2021. Koalas were captured using the ‘flagging’ method (Madani *et al.*, 2020; Hampton *et al.*, 2022). Once captured, koalas were transported within a portable animal carrier to a processing station within the study site. Welfare checks included thorough observations for physical signs of injury, obvious signs of disease, as well as parasitic ticks and dehydration included.

### Koala health and body condition examinations

Health and body condition assessments were completed by a qualified veterinarian and included a thorough examination for physical signs of injury, obvious signs of disease, including wet bottom, periodontal disease, ocular dysfunction, parasite burdens, body condition score and dehydration estimation. Body condition was scored by feeling (palpating) the muscles overlying the shoulder bone (scapula). Body condition was scored between 1 and 5, where 5 is excellent, 4 is good, 3 is fair, 2 is poor and 1 is emaciated. A koala with excellent body condition is defined by strong muscle tone, with the scapula spine palpable on careful palpation, and convex muscle mass on either side of scapula. Conversely, a koala with emaciated condition has noticeable dishing of muscles on either side of scapula, and at times almost no muscles palpable on either side of scapula.

### Sample collection

Koalas were anaesthetized using a combined intramuscular injection of 0.04 mg/kg of medetomidine hydrochloride (Ilium medetomidine, 1 mg/ml, Troy Laboratories, NSW, Australia) and 2-mg/kg alfaxalone (Alfaxan, 10 mg/ml, Jurox, NSW, Australia). On conclusion of the clinical procedure 0.16 mg/kg of atipamezole (Ilium atipamezole, 5.0 mg/ml, Troy Laboratories, NSW, Australia) was administered intramuscularly by a qualified veterinarian to reverse the medetomidine and accelerate recovery from the sedation. A 2 cm × 2 cm section of fur covering the cephalic vein was shaved for blood collection with a 5 ml syringe and 23-g, 5/8-inch needle. The blood was stored in EDTA tubes for manual blood smear analysis and automated haematology analysis, and plain serum tubes for biochemistry. Five to ten fresh faecal pellets were collected from the koala pet carrier following sedation. Additional faecal samples were also massaged from the cloaca of the koala and placed in Ziplock bags and stored at −20°C within 8 hours of collection. Of the 10 captive koalas in the study, faecal samples were obtained upon examination for four individuals, and the faecal samples for the other six were collected 5 days later within enclosures immediately after defecation. No effect of day of faecal sampling was detected during statistical analysis. 

### Haematology analysis

The Sysmex xn-1000 was used to analyse each koala blood sample (Sysmex XN1000, IDEXX Laboratories, Adelaide). The Sysmex xn-1000 uses fluorescent flow cytometry to deliver total WBC count, red blood cell count and WBC differentials. The abundance of neutrophils and lymphocytes as percentages of total WBC was used to calculate the N:L ratio.

### Faecal glucocorticoid metabolite EIA

The analysis of faecal cortisol metabolites was carried out using polyclonal R4866 cortisol enzyme-immunoassay, which has been previously validated for koalas (Narayan *et al.*, 2013). Concentration of FCMs was determined using a polyclonal anticortisol antiserum (R4866) diluted 1:15 000, horse-radish peroxidase conjugated cortisol label diluted 1:80 000 and cortisol standards (1.56–400 pg.well^−1^). Samples were assayed on Nunc Maxi-Sorp plates (96 wells) and in duplicate. Plates were read at 450 nm (reference 630 nm) on an EL800 (BioTek) microplate reader. Assay sensitivity for FCM EIA was 1.04 + 0.20 pg.well^−1^. Intra-assay coefficients of variation were 5.5% and 3.5% for low- and high-percentage-bound controls, and interassay coefficients of variation were 12.1% and 1.5%,Parnell respectively.

The R4866 sample assay reagents (previously supplied by the laboratory of late Prof Coralie Munro at the University of California, Davis, USA) have been used for monitoring FGMs in koalas and other mammals recently and for over 20 years (Wasser *et al.*, 2000, Parnell *et al.*, 2014, Eckardt *et al.*, 2016, Murray *et al.*, 2020). Our research group conducted the first biological validation of the polyclonal R4866 Ab in koalas using an ACTH challenge (Narayan *et al.*, 2013), and since then, this validated assay has been used to test research questions related to koala ecology, conservation physiology and welfare published in numerous studies (Narayan, 2019).

Challenges exist with accurate detection and interpretation of hormone metabolite concentrations in faeces, including the potential for cross-reactivity of EIA and the effects of diet, sex and genetic variation in metabolism (Goymann, 2012; Pribbenow *et al.*, 2017).Our intent in this study was not to pin-point the exact glucocorticoid metabolite captured by the polyclonal R4866 cortisol Ab EIA, which captures a broach spectrum of faecal glucocorticoid metabolites, but to represent the data as an overall measure of faecal cortisol metabolites (FCMs) to compare individuals within the population.

### Statistical analysis

All statistical analyses were performed using the *R* statistical programming software (Version 4.1.1, R Core Team, 2021). We analysed two different sets of models, one to examine variation in FCM concentrations as a response variable, and another to examine variation in neutrophil % as a response variable. We used an information-theoretic approach to compare candidate sets of statistical models that included different combinations of fixed factors that we expected to have an association with variation in FCM concentrations or neutrophil %, respectively. We compared models using Akaike’s Information Criterion (AIC), which was calculated using the number of fitted parameters (*k*), including the intercept, in the model, and the maximum likelihood estimate (*L*) for each model as follows (Symonds and Moussalli, 2010):


$$ AIC=-2\ln (L)+2k $$

We then applied the sample size correction to our AIC values that is recommended for sample sizes *n* < 40 using the following equation:


$$ {AIC}_c= AIC+\frac{2k\left(k+1\right)}{n-k-1} $$

Each model from a candidate set of models is ranked by *AIC_c_*, with the best approximating model being the one with the lowest AIC*_c_* value. The AIC*_c_* thus considers how well the model fits the data (based on the model likelihood), but models with greater numbers of fitted parameters (*k*) are penalized for the inclusion of extra parameters. In other words, models with fewer parameters are favoured if they have the same or similar likelihoods. We then calculate for each model an Akaike weight (*w_i_*), which is a value between 0 and 1, with the sum of the weights of all models compared being 1, and the better fitting models having higher weights than poorer fitting models. We calculated AIC*_c_* values and *w_i_* using the ‘aictab’ function in the ‘*car’* package v3.12 (Fox and Weisberg, 2019) in *R*.

### FCM concentrations

For FCM concentrations, we started by fitting an intercept-only model with no fixed factors against which we compared our candidate models. We then fitted three simple models that each included one of the following factors: captivity status, sex or body condition. We fitted a model with all three main effects in an additive model. We also fitted a model that included an interaction between captivity status and sex as a test of the hypothesis that wild males are under greater stress compared to wild females. We limited the complexity of our models because with a sample size of *n* = 30, including all possible main effects and interactions would have been spurious.

We also sought to assess whether FCM concentrations were associated with N:L ratios. Despite being common practice, the direct inclusion of ratios and percentages as fixed factors in statistical models is not recommended because parameter estimates are biased by the dispersion in the denominator (Packard and Boardman, 1988; Curran-Everett, 2013). To account for variation in neutrophil abundance associated with variation in total WBC abundance, we fitted total WBC count as a covariate in our model. Then, following the approach of (Kronmal, 1993) for the specification of ratios in linear models, we included the main effects of neutrophils (N) and the *inverse* of lymphocytes (L^−1^), and their interaction, with N × L^−1^ being mathematically equivalent to $\frac{N}{L}$.

**Figure 1:**
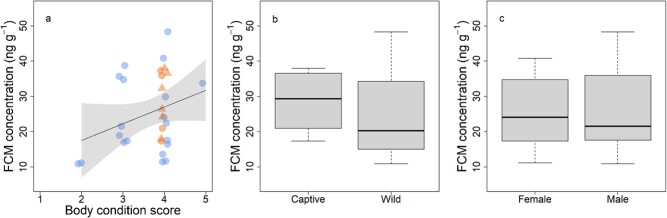
Variation in FCM concentrations in koalas on Kangaroo Island, showed a weak positive association with body condition score (a); a non-significant trend towards lower concentration in wild compared to captive koalas; and no difference between males and females (c). In (a) wild koalas are represented by the blue circles, and captive koalas are shown in orange circles or triangles, for samples collected on initial day of health check or collected from koala five days later, respectively. The shaded area shows 95% confidence interval of the regression line. In (b) and (c), box plots represent the middle 50% of the data with the median shown by the black line, and the lower and upper whiskers representing 1.5× the interquartile range beyond the first and third quartile, respectively.

**Table 1:** Summary statistics for the variation in FCM concentrations (ng/g) measured in koalas on Kangaroo Island

**Factor**	**Level**	**Minimum**	**Maximum**	**Mean**	**Standard deviation**
Sex	Male	10.89	48.32	22.54	11.84
	Female	13.57	40.79	27.62	9.21
Captivity status	Wild	10.89	48.32	25.53	11.37
	Captive	13.57	40.79	25.20	9.26

### Leukocyte profiles: Neutrophils and lymphocytes

We aimed to examine the influence of captivity status (wild, captive), sex (male, female) and body condition score on the N:L ratio. As described above, the use of ratios or percentages as variables in linear models can lead to incorrect inferences (Packard and Boardman, 1988; Kronmal, 1993; Curran-Everett, 2013), so we approached the question by specifying models that contain a parameter that represents a change in the N:L ratio with our factor(s) of interest. To do this, in each model we fitted lymphocyte count as a continuous variable in the model because the abundance of lymphocytes generally decreases as the abundance of neutrophils increases. We then fitted three separate models, each with an interaction between lymphocyte abundance and a factor of interest, that is, captivity status, sex or body condition. An interaction between lymphocytes and our factor of interest would mean that as neutrophils increase and lymphocytes decrease, they do so more steeply in one group compared to the other group. In other words, the interaction term in our models reflects a difference in the rate of change in the N:L ratio in one group compared to the other. We also fitted three additive models, each with lymphocyte abundance and one factor of interest (captivity status, sex or body condition) but no interaction. These models represent the situation where the N:L ratio does not interact with our factors of interest, but that overall, there is a difference in neutrophil count independently of any variation in lymphocyte abundance. In all models, we also fitted total WBC count as a covariate to account for variation in neutrophil abundance associated with difference in total WBC.

## Results

### Koala population density

We estimated the koala population in the 108-ha study area to be 23 koalas, at a population density of 0.21 koalas per hectare. Of the 23 koalas at the study site, only four were koalas that had been translocated into the site in the previous year. We did not observe a single koala carcass at the release site despite extensive surveys of the entire 108-ha area.

### Koala health and body condition

Most of the koalas in our study showed no overt clinical signs of ill health and were in fair (score 3) to good (score 4) body condition (Figure 1a). Diagnostic examination of a range of blood biochemistry parameters also showed little evidence of systemic disease in the koalas in our study (Table S1).

### FCM concentrations

The concentrations of FCM varied between 10.89 and 48.32 ng/g overall. The variation in FCM concentration largely overlapped in males and females, and for captive and wild koalas (Table 1; Figure 1). None of the set of candidate statistical models provided a good fit to the data (Table 2), with the best supported model having an *R^2^* of only 0.09 and *F*_(1, 28)_ = 2.70, *P =* 0.11. The two simple models including body condition or captivity status as a single factor had the same level of support as a model including only an intercept (Table 2). In other words, FCM concentrations did not differ between captive and wild koalas *F*_(1, 28)_ = 1.46, *P* = 0.24 (Figure 1).

**Table 2:** AIC_c_ values (corrected for small sample size) and weights (*w_i_*) for statistical models of FCM concentrations (ng/g)

**Statistical model**	**AIC**_**c**_	***w***_***i***_	**∆AIC**_**c**_	**Log likelihood**
Body condition + ɛ	229.67	0.36	–	−111.37
Intercept only + ɛ	229.95	0.31	0.28	−112.75
Captivity status + ɛ	230.90	0.19	1.23	−111.99
Sex + ɛ	232.31	0.09	2.65	−112.69
Sex + captivity status + body condition + ɛ	234.62	0.03	4.96	−111.06
Sex + captivity status + sex × captivity status + ɛ	235.38	0.02	5.72	−111.44

The change in AIC_c_ between the most supported model and alternative models (**∆**AIC_c_) and the log likelihood for candidate models are also shown. None of the models provided a good fit to the data, with the best supported model having an *R*^2^ of only 0.09 and *F*_(1, 28)_ = 2.70, *P =* 0.11. The ɛ symbol specifies residual error

There was no evidence of a relationship between FCM concentration and the N:L ratio in our study. Our statistical model of FCM concentrations that accounted for total WBC while testing for an interaction between neutrophil abundance and the inverse of lymphocyte abundance (i.e. the N:L ratio) had an *R^2^* of only 0.064 with *F*_(11, 25)_ = 0.43 and *P* = 0.79.

### Leukocyte profiles: Neutrophils and lymphocytes

**Figure 2:**
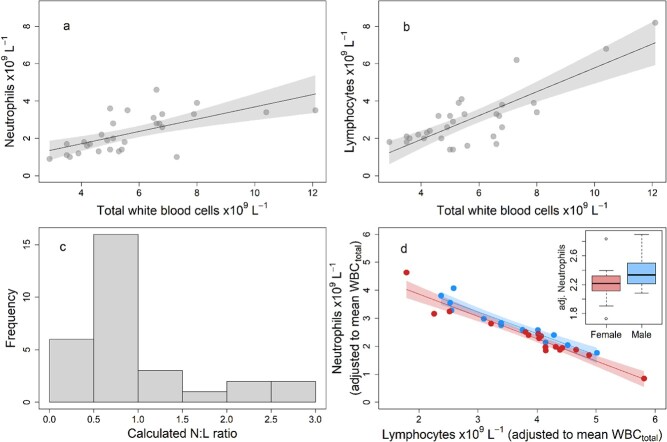
Leukocyte profiles in koalas on Kangaroo Island. Neutrophils and lymphocytes both have a positive relationship with total WBC abundance (a, b). The neutrophil-to-lymphocyte ratio (c) is often calculated from blood cell counts standardized to a percentage of total WBCs and used as a diagnostic marker. In our study, we included leukocyte abundances as variables in statistical models and used the parameters of the model to assess if our factors or interest were associated with variation in the N:L ratio. Show in (d) is the relationship between the abundance of neutrophils and lymphocytes (adjusted to the mean total WBC abundance) in male (blue) and female (red). Shaded areas show 95% confidence intervals for the mean neutrophil abundances predicted by the model. The inset shows the effect of sex on the abundance of neutrophils adjusted to the mean total WBC abundance and to the mean lymphocyte abundance. In other words, the inset visualises the model estimate for the size of the effect of sex on the abundance of neutrophils.

The N:L ratio varied between 0.16 and 2.63 and largely overlapped between the sexes, and between wild and captive koalas (Table 3). Among a set of candidate statistical models of the variation in the abundance of neutrophils (accounting for total WBCs), the model with the most support was an additive model that included the main effects of lymphocyte abundance and sex (Table 4; Figure 2). Importantly, there were no two-way interactions between lymphocyte abundance and captive status, sex, or body condition. The lack of statistical support for the interactive models indicates that there was no difference in the N:L ratio between wild and captive koalas, between the sexes or based on body condition, respectively. The additive effect of sex in the best performing model indicates that there was (weak) support for males have slightly higher neutrophil abundance than females independent of the variation in lymphocytes and total WBCs (estimate 0.18 ± 0.10 s.e., *t*_(26)_ = 1.76, *P* = 0.09).

**Table 3:** Summary statistics for the neutrophil-to-lymphocyte ratio in koalas on Kangaroo Island

**Factor**	**Level**	**Minimum**	**Maximum**	**Mean**	**Standard deviation**
Sex	Male	0.33	2.54	1.03	0.65
	Female	0.16	2.63	0.84	0.64
Captivity status	Wild	0.33	2.63	0.97	0.68
	Captive	0.16	2.01	0.82	0.56

**Table 4:** AIC_c_ values (corrected for small sample size) and weights (*w_i_*) for statistical models of neutrophil %

**Statistical model**	**AIC**_**c**_	***w***_***i***_	**∆AIC**_**c**_	**Log likelihood**
WBC_total_ + lymph. + sex + ɛ	6.51	0.32	–	3.00
WBC_total_ + lymph. + ɛ	7.00	0.25	0.49	1.30
WBC_total_ + lymph. + captivity + ɛ	8.14	0.14	1.63	2.18
WBC_total_ + lymph. + body cond. + lymph. × body cond. + ɛ	8.98	0.09	2.47	3.33
WBC_total_ + lymph. + body cond. + ɛ	9.32	0.08	2.81	1.59
WBC_total_ + lymph + sex + lymph × sex + ɛ	9.53	0.07	3.03	3.06
WBC_total_ + lymph. + captivity + lymph. × captivity + ɛ	10.44	0.04	3.94	2.60
WBC_total +_ ɛ	76.63	0.00	70.13	−34.86

The change in AIC_c_ between the most supported model and alternative models (**∆**AIC_c_) and the log likelihood for candidate models are also shown. The best supported model had an *R*^2^ of 0.953, *F*_(3,26)_ = 176.2, *P* < 0.0001. The ɛ symbol specifies residual error

## Discussion

We found that koalas in areas of remaining unburnt habitat were resilient to the translocation of a large number of bushfire-rescued koalas into their habitat. We had expected to find that population density within the release site would be high due to the influx of 90 translocated koalas. Consequently, we expected that the koalas in the recipient population would show signs of chronic stress and ill health because of increased territorial conflict over food trees and reproductive opportunities. In contrast to our expectations, only one-fifth of the population remaining at the site was translocated koalas, and the overall population density was also much lower than anticipated. Given that we did not observe any deceased koalas at the release site, despite extensive surveys, we think that the majority of translocated koalas dispersed into surrounding habitat areas through existing riparian corridors during the year since the releases had occurred. This finding is similar to a small-scale study from 1998 referenced in Menkhorst and Rylah, 2017) who found that all 20 translocated koalas in the southeast of South Australia dispersed away from the site of release.

Importantly, we observed very little evidence that koalas remaining at the release site were experiencing chronic stress, ill health or physical trauma. Veterinary examinations found that most koalas were in fair to good body condition, with very few diagnostic indicators of systemic ill health. Our stress marker measurements of FCM concentrations and N:L ratios did not differ between the wild koalas and a sample of non-public-display captive koalas and were generally low compared to other studies (Narayan and Vanderneut, 2019; Fabijan *et al.*, 2020b). Overall, our results suggest that, if there is adequate landscape-scale habitat connectivity and opportunity for dispersal, koala translocation has limited impacts on recipient koala populations at translocation release sites. With most existing research on koala translocations (Menkhorst and Rylah, 2017), and in translocation physiology generally (Tarszisz *et al.*, 2014), having focused on the animals being moved, our study makes an important contribution, and is one of the first of its kind to investigate the impacts of wildlife translocations on recipient populations.

The FCM concentrations found in our study were towards the lower end of published values for koalas (Davies *et al.*, 2013b, Narayan, 2019, Narayan and Vanderneut, 2019, Narayan *et al.*, 2013, Santamaria *et al.*, 2021, Webster *et al.*, 2017). We reported a mean of FCM concentrations of 25.42, which is similar to FCM concentrations in healthy, wild koalas from eastern Australian populations with a mean of 12.3 for males, and 6.2 ng/g for females (Narayan *et al.*, 2013). Conversely, in rescued koalas from eastern Australian populations with clinical disease (e.g. Chlamydia) or trauma (e.g. vehicle collision, dog attack, bushfire injury), FCM concentrations have been reported at two to four times higher on average than those in our study, and up to 17 times higher in terms of maximum levels (Narayan and Vanderneut, 2019).

The haematological profiles observed in the koalas in our study suggest that most koalas were in good health. The N:L ratios were at the low end of recently published reference intervals for South Australian koalas (Fabijan *et al.*, 2020b). In that study, which was completed pre-bushfire, the mean N:L ratio was 2.7 times greater than in the present study. We note, however, that for logistical reasons in that study, blood was sampled the day after capture, transportation and overnight captivity of wild-caught koalas. In the present study, blood samples were collected within the hour of capture, which is a short enough time to prevent haematological impacts of the effect of a stress response to capture and handling (Davis and Maney, 2018). The N:L ratios we observed were more similar those observed at time of capture in a different sample of koalas from Kangaroo Island (Hajduk *et al.*, 1992). The total WBC counts and total neutrophil and lymphocyte counts we observed were within published reference intervals for healthy wild koalas from Port Macquarie, NSW (Canfield *et al.*, 1989a; Canfield *et al.*, 1989b).

There are limits to the inferences that can be made from comparisons of the absolute levels of FCM concentration and N:L ratios between the koalas in our study and those in other studies and in other populations. Those limitations arise due to the considerable variation in FCM concentrations and N:L ratios among individuals and populations, across seasons, with age, reproductive and health status and environmental variation (Goymann, 2012; Palme, 2019). For instance, the southern Australian koala populations are a distinct genetic cluster to the eastern Australian populations (Lott *et al.*, 2022), with distinct climatic and habitat variations and differences in the incidence and severity of infectious diseases (Fabijan *et al.*, 2020a). Ideally, we would have been able to compare our results against experimentally validated reference values for our study population. This can be done by measuring FCM concentrations at baseline and after pharmacologically stimulating the HPA axis with an ACTH challenge (Davies *et al.*, 2013b, Narayan *et al.*, 2013), and we suggest that this would be a worthwhile goal of future research.

In lieu of reference values for our specific study population, we compared wild koalas to a sample of captive koalas. Implicit in our comparison is the assumption that the healthy, captive koalas were not experiencing chronic stress. Captivity can bring a range of potential sources of stress, including handling stress, frequent and loud noises, exposure to alien odours, restricted movement, isolation and the formulation of abnormal social grouping (Morgan and Tromborg, 2007; Charalambous *et al.*, 2021). A previous study compared the FCM concentrations of captive and wild koalas and found that the effects of captivity depended on sex and whether or not koalas were handled; handled male koalas had 200% higher FCM levels than their non-handled counterparts, and there was no difference between captive and wild koalas (Narayan *et al.*, 2013). Other studies find evidence that captive koalas that are on public display for photography sessions also experience increased stress (Webster *et al.*, 2017). The population of captive koalas within this study had been kept in cages away from the public, only experiencing low levels of keeper interactions, reducing the potential for unfamiliar human interactions to contribute to their stress levels. Furthermore, 6 of the 10 captive koalas sampled were brought in as joeys with rescued mothers during the bushfires. Those joeys were uninjured and were raised by keepers from a young age, leading to habituation to the presence and handling of the keepers. Hence, we expected that the captive reared group would have lower stress levels than the wild koalas. Additionally, as the captive koalas were kept in a rural area of Kangaroo Island, the influence of loud noises that may influence stress levels would have been reduced compared to koalas in zoos or wildlife parks in more urban areas.

We found no sex-related differences in the variation in the stress markers we measured. Our results concur with a recent study that found no difference between males and females when measuring specifically cortisol concentrations in koala faecal samples (as we did in our study) but did detect a sex difference when measuring other glucocorticoid metabolites (Santamaria *et al.*, 2021). Other studies have found that males had higher FCM concentrations than females (Narayan *et al.*, 2013; Webster *et al.*, 2017).

In conclusion, our study has examined the physiological impacts of wildlife translocation from the perspective of the recipient population. For territorial species, such as the koala, stress associated with the arrival of newly translocated individuals might be short-lived and potentially beneficial if it mediates the successful defence of territory. It is also important to note, however, that the outcome of wildlife translocations, for both translocated and resident animals, will depend on the availability and configuration of habitat in the landscape.

## Funding

This work was supported by a grant from the Kangaroo Island Wildlife Park [CNTR0012650 to KBdS and JB].

## Data Availability

The data and R code underlying this article will be made available upon request via email to corresponding author.

## Supplementary Material

Web_Material_coac088
