# Supplementary material for: Resident wild koalas show resilience to large-scale translocation of bushfire-rescued koalas
Source: Conserv Physiol. 2023 Jan 28;11(1):coac088. doi: 10.1093/conphys/coac088 (PMC9885738; doi:10.1093/conphys/coac088)
Supplement: Web_Material_coac088 [file web_material_coac088.pdf]

Supplementary Information

Table S1. Health summary for koalas in the study. Blood biochemistry parameters that were higher or lower than the reference intervals are reported in the table. Note, the observation of parameters outside the reference intervals do not, in and of themselves, indicate ill health. A diagnostic assessment by a qualified veterinarian was made by the total results (blood biochemistry, external clinical examination). Abbreviations used in the table are ALP (alkaline phosphatase), MCHC (mean corpuscular haemoglobin), MCV (mean corpuscular volume).

ID	Name	Captivity Status	Age	Body Condition	Health Comments	Blood Biochemistry (High)	Blood Biochemistry (Low)
1	Greta	Captive	2-3	4		ALP	Protein, Globulin
2	Percy	Captive	2-3	4		MCHC	Chloride
3	Houdini	Captive	2-3	4		Bicarbonate	
4	Grace	Captive	2-3	4		Anion gap, phosphorus, lymphocytes, white blood cells, sodium, potassium, anion gap, ALP	Protein, Chloride, MCHC
5	Louis	Captive	2-3	4	Tapeworm fragment found in faeces 01/06/2021	MCHC, Monocytes, potassium, ALP	MCV, creatinine, protein, albumin
6	Minnie	Captive	Sub-Adult	4		ALP	MCHC, phosphorus, protein, albumin
7	Larry	Captive	2-3	4		MCV, lymphocytes, calcium, potassium, ALP	
8	Monty	Captive	4	4		Potassium	Protein
9	Bluey	Captive	8-12	4	Unwell and unsteady for majority of 2021 with blood returning odd results two weeks prior to sampling.		MCHC
10	Mia	Captive	>15	4		Potassium and ALP	Creatinine and Urea
11	Aoife	Wild	5-6	4		ALP	Protein
12	Hermione	Wild	4	3		Potassium	Creatinine
13	Marnie	Wild	2-3	4		ALP	

For Review Only

14	Sam	Wild	Sub Adult	4	Tapeworm	Lymphocytes, potassium,	Platelets
15	11D70	Wild	4	4			Creatinine
16	Dolores	Wild	6-8	3			Creatinine, protein
17	Magic Mike	Wild	4	5		Haematocrit, potassium, ALP	Haemoglobin
18	Tony	Wild	4	4		ALP	Platelets, protein
19	Winky	Wild	6-8	3		ALP	
20	Randy	Wild	2-3	3	Tapeworm	MCHC, potassium	RBC, haematocrit, protein, albumin, creatinine
21	Connor	Wild	4	4			
22	Rocky	Wild	2-3	4	Tick found above right ear	Potassium	Platelets
23	Tanya	Wild	4	4		Phosphorus, potassium, ALP	
24	Tessa	Wild	4	4			MCHC
25	Arnie	Wild	4	3		Monocytes	Protein
26	Bruce	Wild	6-8	2		ALP	
27	Claire Bear	Wild	2-3	4		Potassium	
28	Luna	Wild	5-6	3			Protein
29	Oisin	Wild	4	3	Old scratch on his nose.	Anion gap, ALP	MCHC, chloride
30	Shakira	Wild	6-8	2	Old fracture on her leg with poor renal function.	Chloride	Phosphorus, sodium, anion gap, protein

For Review Only
